# Supplementary figures and images for: Co-transmission of neuropeptides and monoamines choreograph the C. elegans escape response
Source: PLoS Genet. 2022 Mar 3;18(3):e1010091. doi: 10.1371/journal.pgen.1010091 (PMC8932558; doi:10.1371/journal.pgen.1010091)

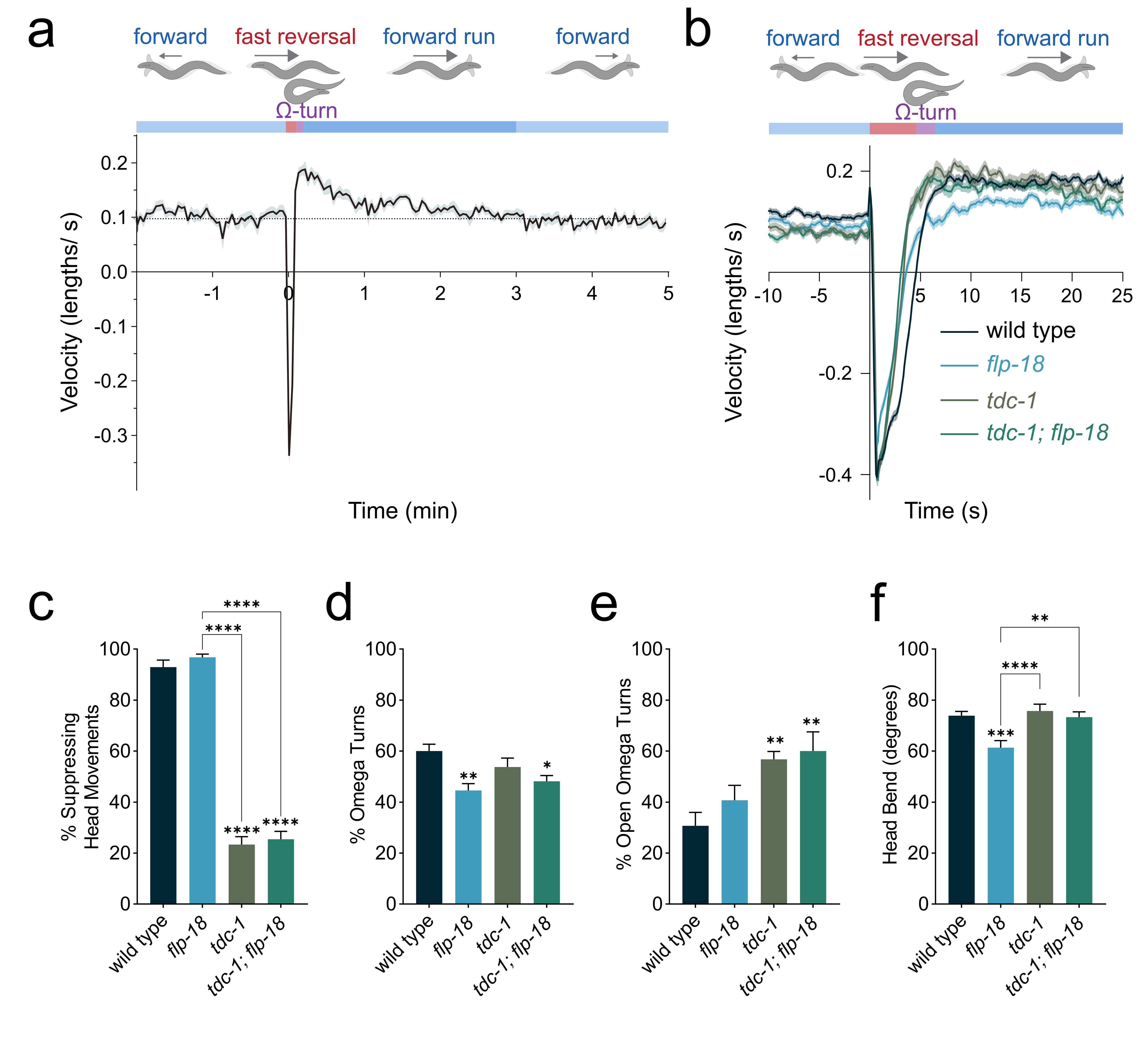

Supplement: S1 Fig — (A and B) Behavioral schematic (top) and velocity traces (bottom) of animals executing the escape response. (A) In wild type animals, forward run velocity remains elevated for approximately 3 minutes after the escape response. Dotted line marks pre-stimulus baseline. (B) Comparison of tdc-1;flp-18 double mutants to the velocity traces presented in Fig 1B. (A-B) Negative velocity indicates backward locomotion. Solid line in trace represents mean velocity, shaded area indicates standard error of the mean. (C-F) Quantification of escape behavior in flp-18, tdc-1, and tdc-1;flp-18 mutant animals. See Fig 3 for details. Graphs represent mean ± SEM, significance was calculated using ANOVA with Šidák’s multiple comparison correction (P<0.05 = *, P<0.005 = **, P<0.0005 = ***, P<0.0001 = ****). Sample size: (A) n = 14 independent trials with 20 worms recorded during each trial. (B) n = # of animals. Wild type, flp-18, and tdc-1 values are reported in Fig 1. tdc-1;flp-18 (n = 204). (C-E), n = # of experiments, 20 worms per experiment. Wild type (n = 12), flp-18 (n = 11), tdc-1 (n = 12), tdc-1;flp-18 (n = 11). (F) n = # of animals. Wild type (n = 88), flp-18 (n = 58), tdc-1 (n = 60), tdc-1;flp-18 (n = 60). (TIF) [file pgen.1010091.s001.tif]

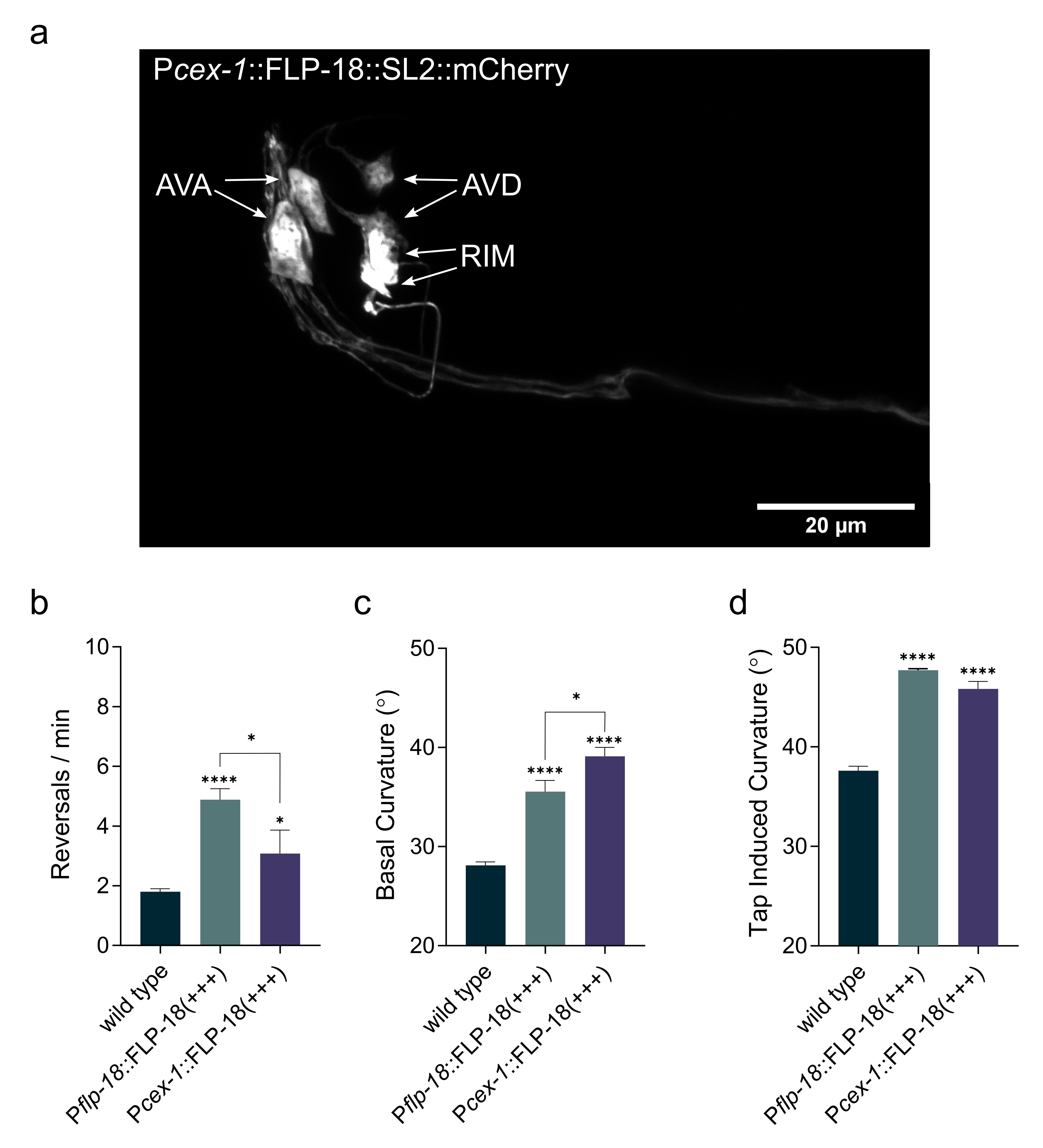

Supplement: S2 Fig — (A) Confocal z-projection of zfEx528[Pcex-1::FLP-18::SL2::mCherry showing detail of head neurons. Cell bodies are labeled, neurons were identified based on anatomical position. (B-D) Analysis of FLP-18 overexpression from endogenous (Pflp-18) and cell specific (Pcex-1) promotors. Spontaneous reversal frequency (B), mean body curvature averaged over 5 seconds prior to (C), or immediately following (D), a tap stimulus. Graphs represent mean ± SEM, significance was calculated using ANOVA with Šidák’s multiple comparison correction (P<0.05 = *, P<0.0001 = ****). Sample size: (B-D) n = # of experiments, 20 animals per experiment. Wild type (n = 16), Pflp-18::FLP-18(+++) (n = 4), Pcex-1::FLP-18(+++) (n = 6). (TIF) [file pgen.1010091.s002.tif]

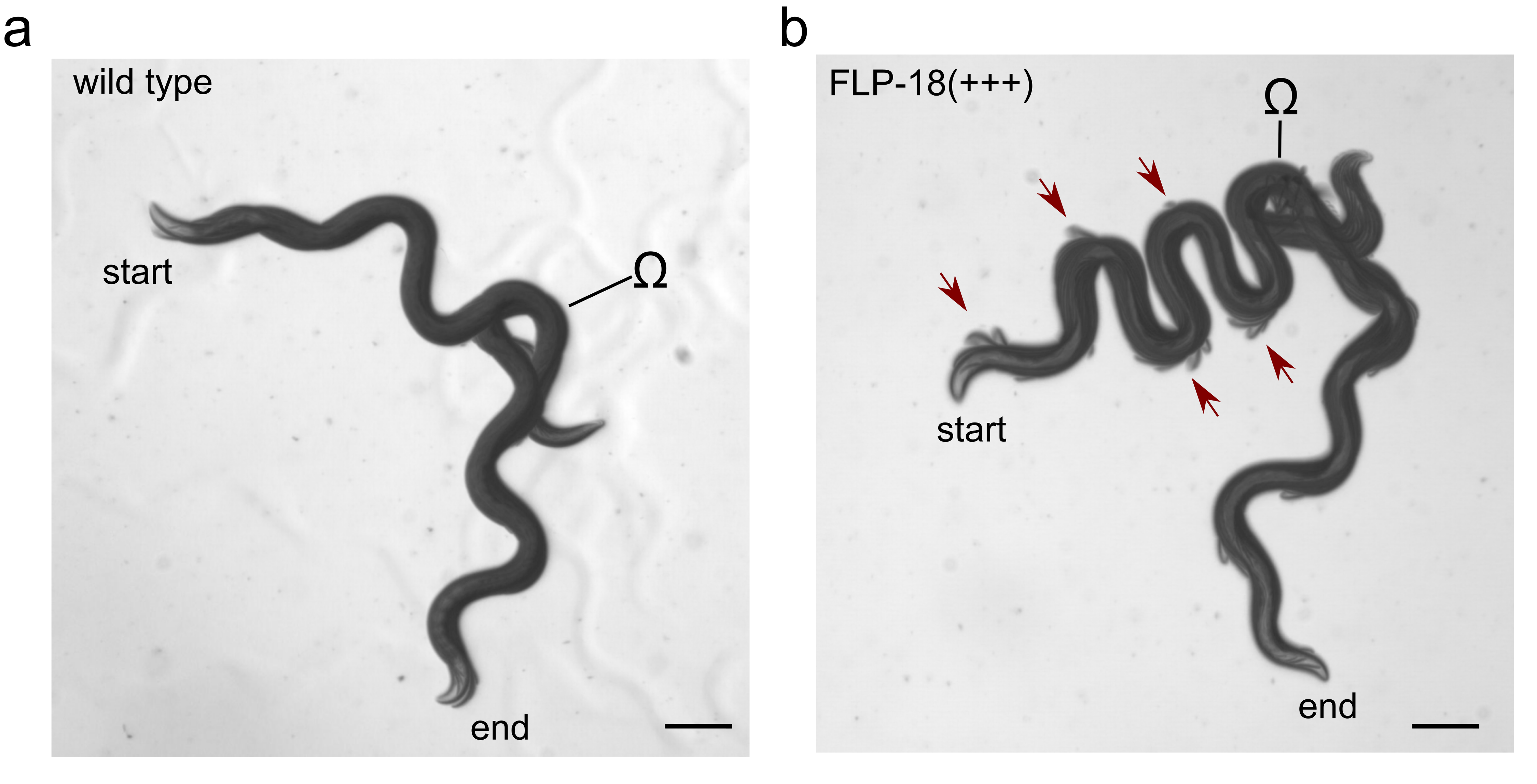

Supplement: S3 Fig — Minimum intensity projections from video recordings of wild type (A) and FLP-18 overexpressing animals (B) after anterior touch. “start” indicates location of head when the animal was touched and initiated a reversal. The omega (Ω) symbol indicates the point where an omega turn was initiated, and the animal switched from reversal to forward motion. “end" indicates the position of the head at the end of the video. The head of the wild-type animal remains relaxed and follows the body during the reversal (A). The FLP-18(+++) overexpressing animal continues head oscillations during the reversal which are visible as the tip of the nose projecting out from the sides of the body and are indicated with red arrows (B). Scale bar = 100μm. (TIF) [file pgen.1010091.s003.tif]

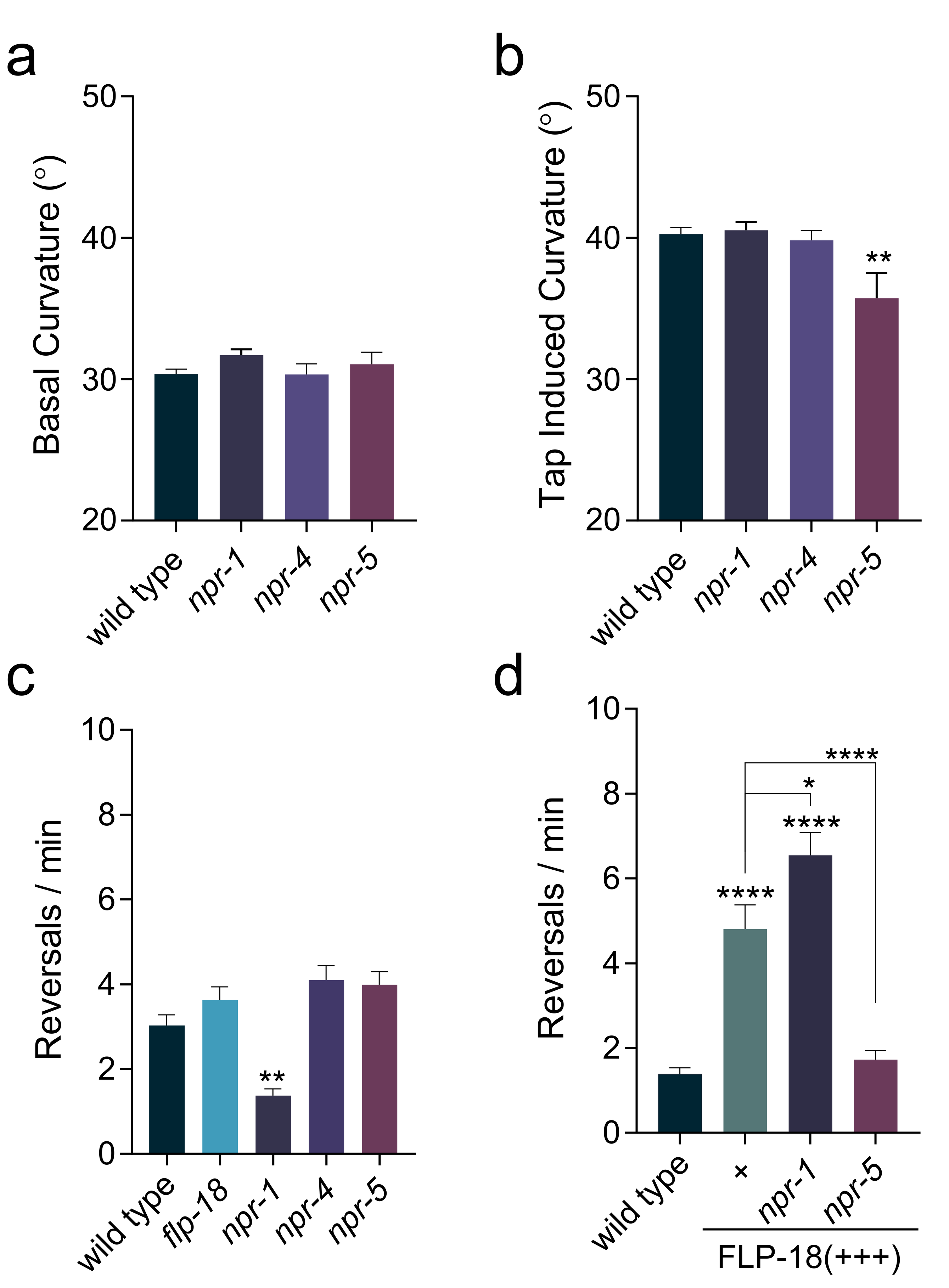

Supplement: S4 Fig — (A and B) Quantification of mean body curvature averaged over 5 seconds prior to a tap stimulus (A) or immediately following a strong tap stimulus (B). (C and D) npr-5 but not npr-1 mutation suppresses high spontaneous reversal frequency in low oxygen conditions. Quantification of spontaneous reversal frequency in ambient oxygen (C) or in low oxygen conditions (D). Mean number of spontaneous reversals per minute per worm averaged over 3 minutes as in Fig 5H. Ambient oxygen experiments (C) were conducted with a standard lid-covered plate in room air. Low-oxygen experiments (D) were conducted on a plate that had nitrogen gas injected into it with a 5ml syringe prior to placing a lid. After lid placement, both conditions were allowed to acclimate for 5 minutes prior to recording. Graphs represent mean ± SEM, significance was calculated using ANOVA with Šidák’s multiple comparison correction (P<0.05 = *, P<0.005 = **, P<0.0001 = ****). Sample sizes (# of experiments, 20 animals per experiment): (A-B) wild type (n = 22), npr-1 (n = 14), npr-4 (n = 12), npr-5 (n = 13). (C) wild type (n = 26), flp-18 (n = 28), npr-1 (n = 14), npr-4 (n = 23), npr-5 (n = 17). (D) wild type (n = 12), FLP-18(+++) (n = 12), npr-1; FLP-18(+++) (n = 15), npr-5; FLP-18(+++) (n = 13). (TIF) [file pgen.1010091.s004.tif]

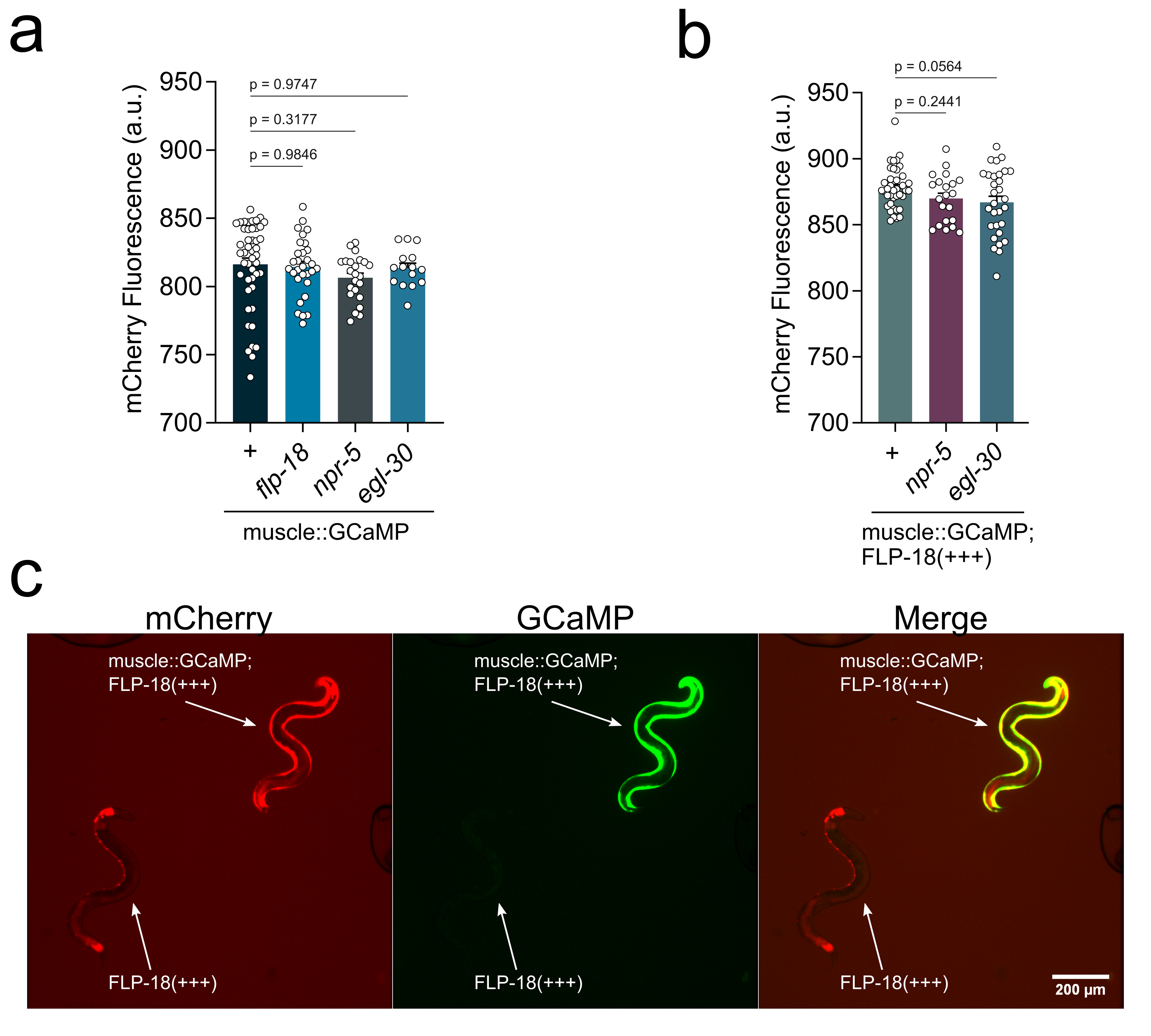

Supplement: S5 Fig — Quantification of mCherry fluorescence in animals expressing the transgene zfEx813[Pmyo-3::NLSwCherry::SL2::GCaMP6] (muscle::GCaMP) in the genetic backgrounds analyzed in Fig 7B. Because the FLP-18 overexpression line (zfIs149[Pflp-18(3kb)::mCherry::SL2::FLP-18]) carries an mCherry transcriptional reporter, mutant lines are compared to their respective control background, muscle::GCaMP (A) or muscle::GCaMP; FLP-18(+++) (B). (C) Fluorescent micrograph showing mCherry, GCaMP and merged signals of a animals expressing zfIs149 alone (bottom, FLP-18(+++)) or in combination with zfEx813 (top, muscle::GCaMP; FLP-18(+++)). Scale bar = 200 μm. Graphs represent mean ± SEM, significance was calculated using ANOVA with Dunnett’s multiple comparison correction (P>0.05 = ns). Sample sizes (# animals): (A) muscle::GCaMP (n = 46), flp-18 (n = 30), npr-5 (n = 23), egl-30 (n = 15). (B) FLP-18(+++) (n = 39), npr-5; FLP-18(+++) (n = 21), egl-30; FLP-18(+++) (n = 30). (TIF) [file pgen.1010091.s005.tif]
