# Supplementary material for: Co-transmission of neuropeptides and monoamines choreograph the C. elegans escape response
Source: PLoS Genet. 2022 Mar 3;18(3):e1010091. doi: 10.1371/journal.pgen.1010091 (PMC8932558; doi:10.1371/journal.pgen.1010091)
Supplement: Attachment — Submitted filename: Rebuttal MA.docx [file pgen.1010091.s011.docx]

We would like to thank all the reviewers for very helpful and constructive comments on the manuscript. We much appreciate the general positive feedback on the manuscript. We have added additional experiments, rearranged the figures, and updated the text.

The main experimental additions include:

1. Analysis of tdc-1:flp-18 double mutants
2. Behavioral analysis of npr-1/4/5 receptor mutants
3. Characterized defects in locomotory arousal of npr-5 mutants
4. Further analysis of our Ca2+ imaging data.

A detailed response to the reviewer comments are found below.

Reviewer comments are in black, our responses are in blue. Thank you for your attention.

**Comments to the Authors:
Please note here if the review is uploaded as an attachment.**

Reviewer #1: The work of Floorman and Alkema dissects the functional contributions of co-transmission to the behavioural and circuit modules underlying a scape response in C. elegans. The Alkema lab and others have previously shown that the scape response is elicited through the interneurons RIM and AVA and that RIM co-expresses tyramine and flp-18. Tyramine acts on neck muscles to suppress head oscillations and on gabaergic motor neurons to disinhibit body wall muscle. In this work, they examine the role that the release of the neuropeptide flp-18 from the RIM interneurons upon touch stimulation plays in locomotion to elicit scape. They show that the touch-induced scape response consists of a locomotion sequence which includes a reversal, a change in direction through an omega turn and the resumption of forward locomotion with a sustained increase in velocity. Mutants lacking the peptide flp-18 do not display the increase in velocity during forward movement at the end of the scape response and display fewer and abnormally open omega turns. Through epistasis experiments where they test several receptor mutants for their ability to supress the phenotype of flp-18 overexpression, they identify npr-5 as the receptor mediating the effects of flp-18 on locomotion. Based on rescue experiments of npr-5 comparing the effects of driving expression with endogenous versus heterologous promoters, the authors propose that flp-18 action on muscle regulates velocity, whereas action on neurons regulates reversal frequency. Finally, they show that flp-18 increases intracellular Ca2+ levels in body wall muscle in a npr-5- and egl-30/Gα q – dependent manner, an effect which (the authors propose) underlies the increase in velocity mediated by the peptide. Together with their previous findings on the regulation of the scape response by tyramine, this work puts forward a model by which co-release of the monoamine and the peptide act synergistically in body wall muscle and antagonistically in neck muscles. Their action on body wall muscles results in an increase in excitability and worm speed whereas their action in neck muscles regulates the suppression and initiation of head oscillations at different steps of the scape sequence.
Co-transmission of neurotransmitters and neuropeptides is a fundamental property of the nervous system which was identified more than 40 years ago (by Thomas Hökfelt and others). Despite its universality, the impact of co-transmission on circuit function and behaviour is till poorly understood. The work of Floorman and Alkema provide a mechanistic logic for how co-transmission can smoothly orchestrate a behavioural sequence and therefore represent an important advance in the field. Furthermore, the parallel regulation of scape behaviour through the co-release of catecholamines and NPY-related peptides in both worm and mammals suggest that a similar logic may be at play.

Mostly, the experiments are carefully done (the behavioural analysis is particularly powerful) and support the conclusions and model proposed by the authors. I only have a few minor comments.

Thank you for the positive feedback.

- A link between flp-18 and the increase in forward speed during scape has been established through analysis of flp-18 mutants in Fig. 1. However, the mechanisms underlying this role of flp-18 have not been elucidated. The authors suggest in the discussion that the increase in body bends and Ca2+ levels/excitability in body wall muscles induced by flp-18 may result in higher velocity, but these more likely underly the quality of omega turns. In addition, flp-18 overexpression does not result in an increase in speed. The authors should make note of this in the discussion to clarify the conclusions.

The reviewer is correct; we cannot make definitive conclusions about the mechanism by which *flp-18* increases forward run velocity. In our model we hypothesize that FLP-18 mediated increase in muscle excitability may contribute to the forward velocity, but we cannot exclude that neuronal mechanisms are in play. *flp-18* overexpression does not increase forward run speed, but this result is difficult to interpret as these animals are severely uncoordinated due to the deep body bends. We have rephrased our description of the model in the discussion to acknowledge this ambiguity (lines 341-347).

- In relation with the above, data for npr-5 mutants should be included in Figure 1.

Yes, this is an important experiment to include. We have conducted additional experiments to examine spontaneous and tap-induced velocity for *npr-5* mutants. For the flow of the manuscript we have included this as part of figure 6 (a-c) as experiments that examine NPR-5 function are presented there. Like *flp-18* mutants, *npr-5* mutants have reduced forward run velocity. This supports our hypothesis that FLP-18 activates NPR-5 to increase forward run velocity.

- Figure 1 shows that there is a defect in the length of tap-induced reversals in flp-18 mutants but this is not mentioned in the text. This is particularly relevant in light of the findings the authors refer to in the discussion (line 320): “Recent reports have shown FLP-18 acts through NPR-4 and NPR-5 in the AVA and sensory neurons to control reversal lengths”

The reviewer is correct; there is indeed a reduction in reversal duration for *flp-*18 mutants. We now included this in the revised manuscript and describe this in the results section (lines 143-144).

- The sentence “FLP-18 neuropeptide since it is the only FRFamide known to be expressed in the tyraminergic RIM neurons and the AVA pre-motor interneurons (Kim and Li, 2004; Rogers et al., 2003)” although correct, it is a bit misleading because both RIM and AVA express other FRFamide peptides. Could the authors modify this sentence to clarify this, and also cite the single cell mRNA profiling paper Taylor et al 2021?

Thank you for pointing out the Taylor paper. We have rephrased the text in results section. We also mention in the discussion that other FLP peptides are expressed in the RIM based on Taylor et al. 2021.

- Line 169 “This indicates that FLP-18 release from the escape circuit neurons AVA and RIM is sufficient to enhance body bending and reversals”. The authors should tone down this conclusion since cex-1 drives (ectopic) expression also in AVD, which is part of the reversal circuit.

We agree. We have added a potential contribution of the AVD to this sentence.

- The conclusion that npr-5 acts in muscle to enhance body bending and through neurons to increase reversal frequency could be strengthened by driving expression of npr-5 under the panneuronal rab-3 promoter. Also, in this results section referring to figure 6, the action of npr-5 in neurons comes a bit as a surprise because neuronal expression is neither mentioned in the text nor seen in the reporter images. The authors should indicate earlier that npr-5 is also expressed in neurons.

Neuronal expression of *npr-5* expression was reported by others and was referenced in the original manuscript, but we failed to clearly present potential neuronal role for NPR-5. Our *npr-5* reporter only expresses faintly in neurons and expression is much stronger in the overlying muscle arms which made it difficult to see cell bodies clearly. We updated figure 6 to include an image where neuronal expression of NPR-5 is visible (indicates by the arrows). Panneuronal expression of NPR-5 will be harder to interpret: ectopic expression in neurons that may affect reversal frequency and NPR-5 can be activated by other FRMF peptides as well. With additional time and neuron specific expression of NPR-5, we could dissect the neuronal requirement of NPR-5 for reversal behavior. The main message of the paper focusses on the effect of NPR-5 on muscle excitability although we do not exclude possibility of neuronal contributions of NPR-5 in the escape response. Given the limited time for the revision we focused on addressing the other comments and toned down our conclusion on the neuronal role of NPR-5 in regulating reversal frequency (line 281-286).

Typo:
- In line 205 “change as a result of tap treatment (Fig. 3f)”. Should say 4f

Thank you, we have corrected this error.

Reviewer #2: In this manuscript, Florman and Alkema characterize the role of FLP-18, a neuropeptide that RIM co-releases with tyramine during the escape response. First, they show that mutants lacking flp-18 fail to enhance their speed after tap-induced reversals, even though several other aspects of the escape response (e.g., likelihood of reversing) are intact in these mutants. They then show that overexpression of flp-18 increases body curvature, and foraging (nose movements). Correspondingly, flp-18 mutants display fewer omega turns and more incomplete omegas during the escape response. They then use Venus-tagged FLP-18 to show that the FLP-18 peptide is released in response to repeated mechanical stimuli (to repeatedly induce the escape response). An analysis of known flp-18 receptors identifies npr-5 as a receptor whose mutation phenocopies flp-18. In addition, loss of npr-5 suppresses the flp-18 overexpression effects on curvature, head movements, and omega turns. Overexpression of npr-5 under its own promoter causes increased tap-induced curvature. Overexpression in muscle causes a basal increase in curvature. Finally, the authors use GCaMP imaging in muscle to show that FLP-18 overexpression causes increased muscle depolarization, dependent on npr-5 and egl-30.

Overall, this is an interesting paper and the experiments are for the most part convincing, especially the genetic/behavioral analysis.

We thank the reviewer for the kind words.

I have a few concerns:

1. The reduction in FLP-18::VENUS signal over hours of repeated tap stimuli is consistent with release of FLP-18 during the escape response, but given that there is no increase in coelomocyte signal, the results seem a bit ambiguous. Could the authors perform this experiment in a unc-31 neuropeptide release-deficient mutant to ensure that the signal change is due to peptide release? Another possible control strain could also be RIM::HisCl to prevent depolarization during tap.

We agree with the reviewer in that we also expected to detect increased fluorescence signal in the coelomocytes after repeated tap stimuli. The reduction in fluorescence in the AVA and RIM is obvious While we do see a trend of increased fluorescence at the 1hr timepoint, but this is no longer visible at the 2 hr time point. Although counterintuitive we did want to include these data in in Fig. 4f. The reason for lack of measurable increase of fluorescence can be twofold: 1) overexpression of FLP-18::Venus under the endogenous promoter may lead to high basal levels of release from other neurons, such as the AIY and RIG which release FLP-18 during foraging. 2) Fluorescence signal is actively broken down in the coelomocytes making it harder to see changes due to. These factors can make any tap-induced increase in coelomocyte fluorescence hard to distinguish. We have previously shown that RIM is activated upon tap stimulation (De Rosa et al. 2019). Together with the reduction in FLP-18::Venus fluorescence in the AVA and RIM, we think that release of FLP-18 is the most straightforward explanation. We agree with the reviewer that the suggested control experiments (with a negative outcome) could contribute to the weight evidence. We hope the reviewer can appreciate that generating the required strains and repeating these experiments would go beyond the given time constraints for the resubmission.

2. The effects of cex-1::FLP-18 expression are only provided in a video format. It would be worthwhile to quantify these results and perform statistical analysis to test whether the results are robust. I would also suggest making and analyzing gcy-13::FLP-18 to specifically assess the role of RIM-released FLP-18. Adding this aspect of cell specificity to the manuscript would strengthen the study.

We have included a supplemental figure 2b-d with statistical quantification of *Pcex-1*::FLP-18(+++) animals in relation to the wild type and the *Pflp-18*::FLP-18(+++) overexpressor.

Additional cell specific experiments could further dissect the role of the AVA vs RIM in FLP-18 release. The gcy-13 promoter leads to lower expression levels in our hands. Therefore we chose the cex-1 promoter which is expressed in both the flp-18 expressing AVA and RIM neurons of the escape circuit. The caveat for any cell specific over expression experiment is the possibility that FLP-18 functions through extra-synaptic, volume transmission, diffusing broadly to affect targets that lack synaptic connections. We do not want to claim that only FLP-18 release from the AVA and RIM affects body bending and reversals, but just that release from the AVA and RIM is sufficient.

3. The muscle GCaMP imaging is interesting, but I’m slightly concerned about the quantification. The authors observe increased muscle GCaMP signal in FLP-18 overexpression animals, but there is no control signal to normalize to. Can the authors perform ratiometric imaging (normalizing the GCaMP signal to a co-expressed red fluorophore)? This would rule out the possibility that flp-18 overexpression simply enhances the GCaMP expression levels.

We agree with the reviewer and added this to the revised manuscript. To address the concern that FLP-18 signaling could affect expression levels of our GCaMP construct we have added a supplemental figure (Fig. S5) quantifying the mCherry fluorescence in the genetic backgrounds presented in Figure 7. We use a myo-3::GCaMP with an SL2 linked mCherry reporter to make sure that *flp-18* does not change GCamp expression. We did not see any changes in the mCherry expression in the different genetic backgrounds. Our FLP-18 overexpression line contains an SL2 linked mCherry transcriptional marker. The presence of this additional cherry transgene made it hard to do direct ratiometric comparisons. At low magnification which visualizes the whole animal, the movement artifacts are much less of a concern. Comparison of mutants to their respective ‘wild type’ backgrounds (muscle::GCaMP vs muscle::GCaMP; FLP-18(+++)) shows no difference in mCherry fluorescence caused by mutations in FLP-18 signaling.

4. Can the authors make a flp-18;npr-5 double mutant to test for additive vs non-additive effects.

NPR-5 can bind other neuropeptides and FLP-18 certainly activates other receptors. We agree in principle that the *flp-18; npr-5* double and e.g., non additive effects would further build the weight of evidence. Our behavioral data show that *npr-5* mutants largely phenocopy the behavior *flp-18* mutants in the escape response. Furthermore, *npr-5* mutants suppress all aspects of FLP-18 overexpression phenotype that we examined. This indicates that NPR-5 it is the major player in mediating the effect of FLP-18 on the escape response. Given the time constraints of the resubmission we focused on the other experiments.

Minor concerns:
1. It would be helpful to cite other examples of co-tranmsmission in C. elegans and compare the models arising from these different studies. One example is serotonin and nlp-3 co-release from HSN.

Thank you for bringing this to our attention. We now have included a comparison of NLP-3/5-HT and other co-transmitter models in the discussion (line 399-408).

Reviewer #3: In the paper “Co-transmission of neuropeptides and monoamines choreograph the C. elegans escape response”, the authors show that release of FLP-18 during touch-evoked reversals acts through NPR-5 to increase intracellular calcium release in the neck and body muscles. This, in combination with tyramine release from RIM, enhances the overall escape response through increased bending of the head and the body. The authors show that overexpression of FLP-18 results in increased omega turn closure rates and head oscillations, and that this is blocked by npr-5 mutation. Overall, this is an interesting paper that presents a rather complete genetic pathway but a few additional experiments could be added to help support their claims.

We thank the reviewer for the positive feedback.

Major revisions:
• The order in which data are presented in the figures is at odds with the flow of the main text of the manuscript. Rather than the manuscript walking through each figure, references to each figure occur in many parts of the paper. This makes the manuscript somewhat cumbersome to read, so I would recommend reorganizing the data presentation in the figures to match the logic of the manuscript.

We much appreciate the comment. We extensively reorganized the data presentation to make the flow of the main text and figures more consistent to minimize switching between figures. In some instances we organized the figures by experiment rather than duplicating panels while trying to preserve the logical flow of the manuscript.

• The authors emphasize in the title as well as the introduction/discussion the novelty of elucidating the various, sometimes opposing, roles of co-transmission of neuropeptide (FLP-18) and monoamine (tyramine). However, the bulk of their data only addresses the contribution of FLP-18 to escape behavior and discussion of tyramine is limited mostly to literature references. Data addressing tyramine can be found in figure 1, where a tdc-1 mutant seems to complete its reversal faster than wild-type (Fig. 1a) but does not show a difference in reversal velocity or forward run velocity (Fig. 1c-d). This is in contrast to the flp-18 mutant, which has decreased velocity in both reversal and forward run. These data would imply that FLP-18 and tyramine release both work in separate pathways, at least in this aspect of the escape behavior. I would like to see some data regarding the involvement of tyramine signaling in their other assays to demonstrate its interaction with FLP-18. For example, does a tdc-1; flp-18 double mutant behave more like tdc-1 or flp-18 single mutant in terms of open omega turns and head movement? Does a tdc-1 mutation affect muscle calcium levels? If they find tdc-1 phenotypes in their assays, can they be rescued by RIM expression?

We agree that the characterization of the *tdc-1; flp-18* double is an important in the context of co-transmission. We have included a more thorough analysis of *tdc-1, flp-18* and the *tdc-1;flp-18* double (velocity, omega turning, suppression of head oscillations and ventral head angle) as part of supplemental Figure 1. We have also updated our model (now figure 8) to more clearly lay out how we propose each co-transmitter impacts behavior, in addition to their cellular and molecular targets. The reviewer is correct that the main focus of the paper is on the role of FLP-18 in the escape response. Previous papers from our lab have characterized the role of tyramine signaling on head oscillations, reversal, muscle electrophysiology (Piri et al, 2009, Pirri et al, 2015) and omega turning (Donnely, 2013).

• The authors mention a few times that the forward run velocity data represents a change in the internal state of the animal, and represents a state of arousal. Their evidence for this is the ~3 minute increase in velocity post-reversal, which they call a “forward run”. However, this specific behavior is not investigated in most of the later experiments with FLP-18 overexpression or npr-5 mutation. I would be interested to see if FLP-18 overexpression increases either the velocity or duration of the forward run.

We agree that we should have included forward run analysis of npr-5 mutants (See comment to reviewer 1). We have conducted additional experiments assessing the forward run in *npr-5* mutants (Fig. 6a-c) which show a similar defect to *flp-18* mutants. Overexpression of FLP-18 results in a uncoordinated loopy locomotion, making the impact on the forward run hard to dissect.

• Figure 5 explores possible FLP-18 receptors to determine which one is acting in their behavioral pathway. The authors demonstrate that FLP-18 overexpression phenotypes can be blocked by npr-5 mutation. However, none of their data includes single npr-5 mutant behavior without FLP-18 overexpression. This is important to illustrate the nature of their interaction, and how much the npr-5 mutation contributes to the phenotype alone.

We agree that these are an important set of experiments. This might not have obvious in the original manuscript but some of these experiments were in figure 3f and 3g and supplemental figure 4a. As suggested reviewer we rearranged the figures (now Fig 5 a,b, Supplemental figure 4c) to make this more clear. Furthermore we have added new additional experiments quantifying body curvature in receptor mutants (supplemental figure 4a-b).

Minor revisions:
• The figure references are inconsistently formatted (e.g. 217: “Fig. 3, f”, vs. 225: “Fig. 5d” )

Thank you. We have corrected this.

• Lines 114-115 read: “wild-type animals exhibited a markedly elevated forward locomotion rate for approximately 3 minutes (Fig. S1) – a behavior we termed the ‘forward run’”. However, the figure 1 legend, lines 778-779, states: “(d) Mean locomotion velocity during the forward run phase (t = 7-10s)”. Clarify whether “forward run” refers to either a three-minute period or a 7-10s period.

Thank you; this may have been unclear. We updated both the text of the results section and the figure legends to indicate that we are measuring a time window within the forward run for the statistical comparisons, rather than the longer behavioral state.

• Figure S2b is missing, and it appears that the figure legend for S2b actually refers to S2a, which is the only panel pictured.

We have corrected this.

• Lines 226-228 read: “the proportion of open omega turns made by npr-5; FLP-18(+++) animals is significantly increased and similar to the npr-5 single mutants (Fig. 5e)”. While this trend is as stated, the % open omega turns in Fig. 5e by the npr-5; FLP-18(+++) animals is ~65% but in Fig. 3g, the % open omega turns for the npr-5 single mutants is much lower, at around 40%. The WT response remains at around 20% for both graphs.

This is a sharp observation from the reviewer. All of our behavioral experiments are performed with wild type internal controls and are repeated across multiple days.. We do see some degree of variability in these types of sensitive behavioral assays. This is likely due to the impact of fluctuations in ambient temperature and humidity that affect behavior and is not uncommon.

• In figure 7a, I would be interested in seeing the calcium concentration in body wall muscles that oppose each other in the sinusoidal wave, since one side is contracted and the other, relaxed. With this kind of analysis the authors could see if FLP-18 overexpression increases the calcium concentration but the differential concentration between opposing sides is maintained, or if calcium concentration is increased to the extent that the differential is reduced. The images could also be presented with a colored heatmap representing calcium concentration instead of grayscale to increase the dynamic range of the representative images, which on my screen appear to be saturated in spots.

We do indeed see in moving animals that anterior posterior waves of calcium propagate along the contracted side of the animal. We agree with the reviewer that it would be very interesting to correlate body postures with ventral and dorsal calcium dynamics and differentials. This detailed analysis however is beyond the scope of the main message of this manuscript.

Using a heat map for our Ca^2+^ images is a great idea. We have updated figure 7 to improve the dynamic range. The FLP-18(+++) animal still seems saturated due to its relative brightness but all images were acquired at exposure levels that ensured no saturation of the raw images.

• In the Molecular Biology and Transgenics section, clarify how the high-expressing vs. low-expressing FLP-18 lines were made. Adding plasmid concentrations to the strain list would help with this.

We have added injection concentrations for all our constructs. (high-expressing (200ng/µl vs. low-expressing 20ng/µl).

• Lines 400 – 402 read: “FLP-18 transgenic strains were created by amplifying a 5.8kb sequence of genomic DNA beginning 3.3kb upstream of the open reading frame and containing the flp-18 coding region as well as the 3’ UTR. The extrachromosomal array was stably integrated…”. Please indicate specifically that this region of DNA was cloned into a plasmid and injected. As is it sounds like the amplified PCR fragment was injected. The same applies to the NPR-5 transgenic process.

Thank you for pointing this out. We have revised the molecular biology and strain list to describe our cloning approach, what primers were used, and at what concentrations plasmids were injected.

**Have all data underlying the figures and results presented in the manuscript been provided?**
Large-scale datasets should be made available via a public repository as described in the *PLOS Genetics* [data availability policy](https://nam10.safelinks.protection.outlook.com/?url=http%3A%2F%2Fjournals.plos.org%2Fplosgenetics%2Fs%2Fdata-availability&data=04%7C01%7CJeremy.Florman%40umassmed.edu%7Cbf74c6be404d471f460508d9a90abbe2%7Cee9155fe2da34378a6c44405faf57b2e%7C0%7C0%7C637726685829451347%7CUnknown%7CTWFpbGZsb3d8eyJWIjoiMC4wLjAwMDAiLCJQIjoiV2luMzIiLCJBTiI6Ik1haWwiLCJXVCI6Mn0%3D%7C3000&sdata=n7MeOTWn%2BbvVp0lXtQSY3YLh4wfAzfnVTRLLKDluWe0%3D&reserved=0), and numerical data that underlies graphs or summary statistics should be provided in spreadsheet form as supporting information.

Reviewer #1: Yes

Reviewer #2: Yes

Reviewer #3: Yes
